# Efficacy and procedural efficiency of mechanical thrombectomy devices in posterior circulation stroke

**DOI:** 10.3389/fneur.2025.1620092

**Published:** 2025-07-24

**Authors:** Linlin Ma, Zhe Cheng, Gary B. Rajah, Ho Jun Yun, Xiaokun Geng, Yuchuan Ding

**Affiliations:** ^1^Department of Neurology and Stroke Center, Beijing Luhe Hospital, Capital Medical University, Beijing, China; ^2^Department of Neurosurgery, Munson Medical Center, Traverse City, MI, United States; ^3^Department of Neurosurgery, Munson Healthcare, Traverse City, MI, United States; ^4^Department of Neurosurgery, Wayne State University School of Medicine, Detroit, MI, United States; ^5^Luhe Institute of Neuroscience, Capital Medical University, Beijing, China

**Keywords:** acute ischemic stroke, endovascular therapy, penumbra aspiration catheter, solitaire retriever stent, trevo retriever stent, prognosis

## Abstract

**Background and purpose:**

Posterior circulation stroke patients have worse outcomes after mechanical thrombectomy (MT) and higher mortality than anterior circulation acute ischemic stroke (AIS) patients due to large vessel occlusions (LVOs). To determine the ideal recanalization device for posterior circulation LVO strokes, this study compared the operational parameters and prognosis among three commonly used thrombectomy devices.

**Methods:**

A total of 99 patients with posterior circulation AIS who underwent mechanical thrombectomy were enrolled. The patients were divided into three groups based on the different thrombectomy devices used during the procedure. Patient demographics, procedural metrics, functional outcomes, and symptomatic intracranial hemorrhage (sICH) were assessed. Any association between the devices and favorable clinical outcomes was assessed by logistic regression analysis.

**Results:**

A total of 80 patients were analyzed. The Penumbra aspiration catheter revealed a significant advantage for the time of recanalization vs. the other devices (32 min vs. 44 and 41 min). No significant difference was observed in other procedural parameters or functional outcome. There was no significant difference in symptomatic cerebral hemorrhage (sICH), mortality, or functional independence after MT among the three groups. Diabetes mellitus, NIHSS score at admission, time from onset to recanalization, and occlusion site were associated with functional independence at 90 days, though the use of different recanalization devices did not make a significant difference.

**Conclusion:**

Aspiration achieved vessel recanalization faster than the retriever stent during mechanical thrombectomy in posterior circulation AIS. No clear improved functional outcome favored one device over another in this study. The key factors affecting functional outcomes in posterior circulation LVOs were the presence or absence of diabetes, baseline NIHSS, occlusion site of basilar artery, and TOR time.

## Introduction

Endovascular therapy has improved functional outcomes of patients with acute ischemic stroke (AIS) from anterior large vessel occlusions (LVOs) (1–5); however, data remain less definitive for posterior circulation LVOs. BASICS and BEST randomized controlled trials (RCTs) did not demonstrate significant efficacy of mechanical thrombectomy for basilar artery occlusion. However, the recent BAOCHE study confirmed the effectiveness of acute basilar artery occlusion (BAO) with the Solitaire retriever stent, which provided high-level evidence-based evidence on posterior circulation thrombectomy (6).

As far as we know, not everyone benefits from mechanical thrombectomy (MT), and mortality rates remain higher with posterior circulation stroke despite successful reperfusion achieved via MT (7). Nevertheless, studies have shown that early and complete recanalization are the most important factors for a good clinical outcome for AIS due to vessel occlusions, and mechanical thrombectomy (MT) may be the fastest, safest, and most effective approach (8–12). New thrombectomy devices over the past several years have been improved for efficiency, safety, and distal reach of cerebrovascular recanalization. Penumbra aspiration catheter, Trevo stent retrievers, or Solitaire stent retrievers are commonly used as the second-generation classic thrombectomy devices with governing approval and indications for each. Different devices have been used in previous studies, and few studies have compared the efficacy and safety of the second-generation devices and their impact on prognosis. A meta-analysis including six RCTs (SWIFT, TREVO2, EXTEND-IA, SWIFTPRIME, REVASCAT, and THERAPY) and five study arms (Trevo, Solitaire, Aspiration, Merci, and medical-only) suggests that Trevo or Solitaire retriever stents are more likely to be associated with functional independence, whereas Solitaire retriever stents or Penumbra aspiration catheters appear to be safer for anterior circulation stroke (13). Some current studies have focused on the relationship between thrombectomy technique and prognosis of posterior circulation stroke, but not on different devices and outcomes.

This study retrospectively compared the safety and efficacy of the three second-generation thrombectomy devices in posterior circulation AIS due to LVOs. In addition, prognostic factors associated with posterior circulation stroke and MT were studied.

## Subjects and methods

### Study population

A retrospective analysis of the database of previous cases was performed, which included consecutive patients presenting with acute posterior circulation involving vertebral, basilar, or P1artery. The patients were treated by MT in Beijing Luhe Hospital from 15 June 2015 to 13 June 2021. The inclusion criteria were the followings: (1) age ≥ 18 years; (2) presentation within 24 h from the estimated time of BAO; (3) BAO confirmed by computed tomography angiography, magnetic resonance angiography (MRA), or digital subtraction angiography (DSA); (4) informed consent obtained; and (5) presence of functionally significant symptoms consistent with posterior circulation stroke, regardless of NIHSS score. Patients were excluded from the study in the case of (1) a premorbid modified Rankin Scale (mRS) score >2; (2) brain imaging revealing an bilateral extended brainstem ischemia or large area of bilateral cerebellar ischemia, evidence of intracranial hemorrhage on presentation; (3) a lack of follow-up information; (4) current pregnancy or lactation; (5) a serious, advanced, or terminal illness; (6) incomplete baseline critical data (e.g., imaging and time metrics); and (7) vessel recanalization achieved by other methods (e.g., balloon angioplasty, stenting, alone rt-PA or multiple devices used). Seventeen such patients were retrospectively reviewed for transparency: nine underwent rescue stenting, one balloon angioplasty, two intra-arterial thrombolysis, one mechanical clot disruption using a guidewire, one spontaneous reperfusion, and three received multiple thrombectomy devices. Although all patients achieved successful reperfusion (mTICI≥2b), their 90-day outcomes were heterogeneous (mRS 0–2 in 10 cases, mRS 3–5 in 5 cases, and mRS 6 in 2 cases). These patients were excluded from the main analysis because their procedural outcomes could not be reliably attributed to a single thrombectomy device, and inclusion would have introduced treatment heterogeneity.

### Administration of rt-PA and bridging therapy

Patients who were eligible for intravenous thrombolysis received recombinant tissue plasminogen activator (rt-PA) at a dose of 0.9 mg/kg (10% of the dose was given as a bolus within 1 min, followed by a 60-min infusion) while endovascular intervention was simultaneously being prepared after non-invasive workup. Endovascular procedures were performed under local anesthesia except for agitated or uncooperative patients who were treated with general anesthesia. Interventional strategies were left to the discretion of the treating interventionalists, including the choice of stent retrievers Solitaire FR Device (Medtronic, Minneapolis, MN, United States; commonly 4 × 20 mm, 4 × 40 mm, and 6 × 30 mm) vs. Trevo XP ProVue Retriever (Stryker, Kalamazoo, MI, United States; commonly 4 × 20 mm and 6 × 25 mm) vs. aspiration catheter (Penumbra System 5/4/3MAX Reperfusion Catheters, Alameda, CA, United States), stenting, and other necessary devices. Aspiration thrombectomy was performed using Penumbra Reperfusion Catheters: 5MAX (ID 0.054″, *n* = 11), 4MAX (ID 0.041″, *n* = 5), and 3MAX (ID 0.035″, *n* = 2). The choice of catheter was based on operator preference and vascular anatomy.

### Imaging evaluation

A brain computed tomographic (CT) scan was obtained on presentation as the baseline and immediately after endovascular intervention to assess ischemic stroke burden and ICH. Computed tomography angiography (CTA), magnetic resonance angiography (MRA), or DSA were performed to confirm the presence of acute posterior circulation stroke due to occlusion of proximal (vertebral artery), middle (basilar artery), or distal (posterior cerebral artery) arteries. Magnetic resonance imaging (MRI) was performed 24 h after vessel recanalization. If unable to complete the MRI or any sign of neurological deterioration occurred within 24 h, another CT head scan was performed. Any ICH at 7 days or discharge was recorded and classified into one of the five categories according to the European Cooperative Acute Stroke Study II (ECASS 2) (14). Symptomatic intracerebral hemorrhage (sICH) was defined as radiographic ICH with a ≥ 4-point increase in the NIHSS score from baseline. Target vessel recanalization was assessed by the modified Thrombolysis in Cerebral Infarction (mTICI) scale (15). TICI scoring was performed by two neuroradiologists who were blinded to the specific device used. In cases of discrepancy, a third neuroradiologist was consulted to reach a consensus.

### Neurological status assessments

NIHSS was assessed in the emergency room. Functional outcome was assessed by mRS score; good outcome (i.e., functional independence) was defined as an mRS score of 0 to 2 at 90 days after stroke onset. The mRS score was obtained through an in-person consultation and telephone interview. Death, cause of death, and any systemic bleeding complications were recorded 3 months after stroke onset. The procedural metrics of endovascular therapy were also recorded, including time from onset to recanalization (TOR), time from treatment to recanalization (TTR), and the number of passes of the retriever stent (NOP).

### Statistical analysis

Statistical analyses were performed using SPSS V.24 (IBM Corporation, New York, United States). Patient variables were analyzed using descriptive statistics and univariate comparisons. Comparisons were performed using the Student’s *t-*test for continuous measures, a non-parametric *t*-test for non-continuous variables, and a χ^2^ test for categorical measures. All tests were two-sided, and a *p* < 0.05 was considered statistically significant. Following univariate analysis, multivariate logistic regression was used to assess whether admission NIHSS score, location of stroke (Vertebral, Basilar, P1) could independently predict good outcome (mRS 0–2) or mortality at 90 days after the procedure. Two different models were constructed (good outcome and mortality prediction). Performance of each model was assessed, using the Hosmer–Lemeshow test and the c-statistic. Variables included in these models were age, gender, TOR, baseline NIHSS, IV rt-PA, procedure time, location (basilar, vertebral, P1), comorbidities, complications, good mTICI (2b-3), and NOP. Additionally, occlusion site location (proximal, middle, and distal) was included as a covariate to account for anatomical variability among device groups.

## Results

### Patients

**Figure 1:**
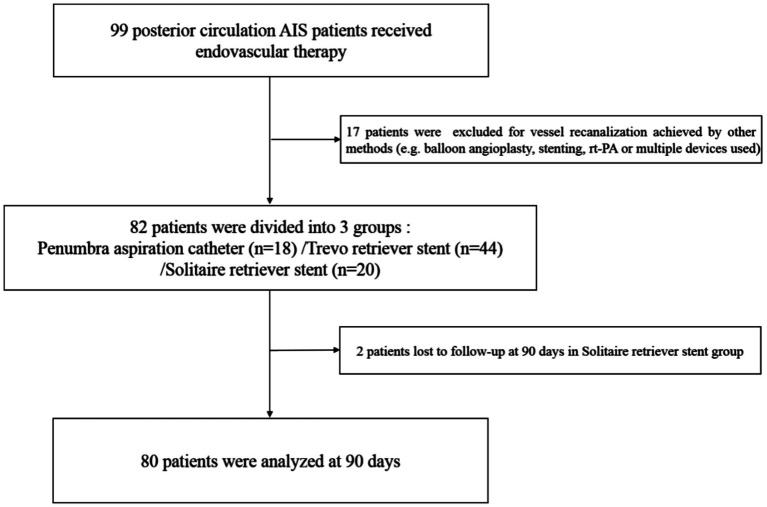
Flowchart of patient selection.

A total of 80 participants were included and analyzed (18 in the Penumbra group, 44 in the Trevo retriever stent group, and 18 in the Solitaire retriever stent group; Figure 1). Baseline demographic and characteristics of the patients are summarized in Table 1. Among the three groups, the proportion of patients with atrial fibrillation in the Trevo group was higher than in the other two groups (*p* < 0.05). Age, sex ratio, baseline NIHSS score, vascular risk factors, and IV rt-PA were not significantly different among the three groups (Table 1).

**Table 1:** Demographic and clinical characteristics of patients.

Baseline variable	Penumbra (*n* = 18)	Trevo stent (*n* = 44)	Solitaire stent (*n* = 18)	*p-*value
Age, mean (SD), y	60.33 ± 10.83	64.23 ± 10.46	64.94 ± 8.38	0.313
Sex ratio (male/female)	17 (94.4)	35 (79.5)	17 (94.4)	0.157
NIHSS, median (IQR), point	23.50 (20–26)	22.0 (16–26)	19 (11–25)	0.212
Risk factors, n. (%)				
Hypertension	15 (83.3)	37 (84.1)	15 (83.3)	1.000
Diabetes mellitus	4 (22.2)	13 (29.5)	5 (27.8)	0.943
Dyslipidemia	5 (27.8)	18 (40.9)	6 (33.3)	0.611
Atrial fibrillation	1 (5.6)	16 (36.4)	5 (27.8)	0.037*
Previous stroke	3 (16.7)	14 (31.8)	6 (33.3)	0.480
Drinking, n (%)	5 (27.8)	13 (29.5)	7 (38.9)	0.804
Cigarette smokers	8 (44.4)	24 (54.5)	13 (72.2)	0.243
Intravenous rt-pa, n. (%)	1 (5.6)	7 (15.9)	3 (16.7)	0.677

NIHSS, the National Institute of Health Scale Score; rt-PA, recombinant tissue plasminogen activator. *p < 0.05.

### Procedural metrics

Among the three groups, mechanical thrombectomy by Penumbra aspiration catheter revealed a significant advantage (*p* < 0.05) on time from treatment to recanalization (32 min vs. 44 and 41 min). There was no significant difference among the three groups in the incidence of basilar artery occlusion, time from onset to recanalization, the number of passes, and TICI classification of the three groups of patients (*p* > 0.05; Table 2).

**Table 2:** Procedural characteristics of patients.

Baseline Variable	Penumbra (*n* = 18)	Trevo stent (*n* = 44)	Solitaire stent (*n* = 18)	*p-*value
Occlusion site, no. (%)				0.116
Proximal	11 (61.1)	15 (34.1)	6 (33.3)	
Middle	7 (38.9)	16 (36.4)	6 (33.3)	
Distal	0	13 (29.5)	6 (33.3)	
NOP, median (IQR)	2 (1–3)	2 (1–3)	2 (1–3)	0.584
TTR, median (IQR), min	32 (25–42)	44 (33–67)	41 (23–66)	0.030*
TOR, median (IQR), min	319 (263–400)	244 (205–355)	326 (182–455)	0.112
TICI score 2b/3, n (%)	15 (83.3)	39 (88.6)	16 (88.9)	0.840

TOR, time from onset to recanalization; TTR, time from treatment to recanalization; NOP, the number of passes of the retriever stent; TICI, thrombolysis in cerebral infarction. *p < 0.05.

### Clinical efficacy and safety outcomes

Compared with the other two groups, the Penumbra group revealed a trend in improving mRS scores at 90 days from stroke onset, but this was not statistically significant. There was no significant difference among the three groups in the proportion of patients who were functionally independent (i.e., mRS 0–2) and mRS 0–3. Additionally, no significant difference was shown among the three devices in mortality and sICH after MT (*P* > 0.05; Table 3).

**Table 3:** Clinical efficacy and safety outcomes.

Baseline variable	Penumbra (*n* = 18)	Trevo stent (*n* = 44)	Solitaire stent (*n* = 18)	*p-*value
mRS at 90 days, median (IQR)	2.5 (0–6)	3.5 (1–6)	3.5(0–6)	0.830
mRS 0–2 at 90 days, n. (%)	9 (50%)	21 (47.7%)	8 (44.4%)	0.945
mRS 0–3 at 90 days, n. (%)	11 (61.1%)	22 (50%)	9 (50%)	0.708
mRS 6 at 90 days, n. (%)	5 (27.8%)	14 (31.8%)	5 (27.8%)	0.926
sICH, n. (%)	0	2 (4.5%)	1 (5.6%)	0.081

mRS, modified Rankin score; sICH, symptomatic intracranial hemorrhage.

### Comparison of baseline characteristics of patients with good vs. poor prognosis related to posterior LVOs

Eighty patients were dichotomized based on functional outcome (good vs. poor) to identify variables to adjust (Table 4).

**Table 4:** Demographic and clinical characteristics of patients between the good and poor prognosis groups.

Baseline variable	Good outcome (*n* = 38)	Poor outcome (*n* = 42)	*p-*value
Age, mean (SD), y	63.84 ± 10.57	63.21 ± 9.87	0.784
Male, n. (%)	31 (81.6)	38 (90.5)	0.334
NIHSS, median (IQR), point	19 (14–24)	24 (19–26)	0.025*
Risk factors, n. (%)			
Hypertension	31 (81.6)	36 (85.7)	0.764
Diabetes mellitus	6 (15.8)	16 (38.1)	0.044*
Dyslipidemia	12 (31.6)	17 (16.7)	0.488
Atrial fibrillation	14 (36.8)	8 (19.0)	0.086
Previous stroke	10 (26.3)	13 (30.9)	0.805
Drinking	14 (36.8)	11 (26.2)	0.342
Cigarette smokers	26 (68.4)	19 (45.2)	0.045*
Occlusion site, no. (%)			0.052
Proximal	10 (26.3)	22 (52.4)	
Middle	16 (42.1)	13 (30.9)	
Distal	12 (31.6)	7 (16.7)	
NOP, median (IQR)	2 (1–3)	2 (1–3)	0.906
Intravenous rt-pa (n (%))	6 (15.8)	5 (11.9)	0.749
mTICI score 2b/3, n (%)	30 (78.9)	39 (92.9)	0.043*
TTR, median (IQR), min	44 (29–60)	54 (39–77)	0.040*
TOR, median (IQR), min	251 (196–324)	328 (212–418)	0.037*

NIHSS, the National Institute of Health Scale Score; rt-PA, recombinant tissue plasminogen activator; NOP, the number of passes of retriever stent; TOR, time from onset to recanalization; TTR, time from treatment to recanalization; mTICI, the modified thrombolysis in cerebral infarction. *p < 0.05.

### Predictors of MT outcome in acute posterior circulation stroke

Logistic regression analysis was performed with the 90-day prognosis as the dependent variable. The variables significant in the univariate logistic regression analysis were included in the multivariate logistic regression analysis. Low baseline NIHSS score at admission, short TOR, and low rate of diabetes mellitus were associated with good prognosis and proximal occlusion with poor prognosis (*p* < 0.05; Table 5).

**Table 5:** Logistic regression analysis of the demographic and clinical characteristics for functional independence.

Variable	Odds ratio	95% CI	*p*-value
NIHSS Score	0.885	0.809–0.969	0.008*
TOR	0.995	0.990–0.999	0.010*
Diabetes mellitus	0.186	0.049–0.707	0.014*
proximal occlusion	0.149	0.034–0.654	0.012*

NIHSS, the National Institute of Health Scale Score; TOR, time from onset to recanalization; *p < 0.05.

### Comparison with BASICS and BEST studies

The major demographic, clinical characteristics, and outcomes of all patients screened in this study were compared with the BASICS and BEST studies. The present study showed better prognostic outcomes with mRS 0–2 percentage (50.5% vs. 35.1% in BASICS and 33.3% in BEST) and mortality rate (24.2% vs. 38.3% in BASICS and 33.3% in BEST). Additionally, shorter vessel recanalization time (280 min vs. 400 min in BEST) and a higher rate of successful recanalization (89.9% vs. 72% in BASICS and 71% in BEST) were noted in this study. Second-generation thrombectomy devices were used in the BASICS and BEST studies, and the same was true in the present study (Table 6).

**Table 6:** Comparison of BASICS and BEST studies.

Characteristic	All patients screened (*n* = 99)	BASICS study (*n* = 154)	BEST study (*n* = 66)
Demographic and clinical characteristics			
Age, mean (SD), y	63.25 ± 10.30	66.8 ± 13.1	62 (50–74)
Sex ratio, no. (%)	77 (77.8)	100 (64.9)	48 (72.7)
NIHSS Score, median (IQR)	21 (15–26)	21	32 (18–38)
IV rt-PA, no. (%)	27 (27.3)	121 (78.6)	18 (27.3)
Basilar artery occlusion, no. (%)	64 (64.6)	NA	59 (89.4)
Procedure of endovascular therapy			
Time from treatment to recanalization, median (IQR), min	47.0 (323–67)	NA	NA
Time from onset to recanalization, median (IQR), min	280 (207–369)	NA	400 (269–526)
TICI≥2b, no. (%)	89/99 (89.9)	63/88 (72)	45/63 (71)
Primary and Secondary Outcomes			
Modified Rankin score of 0–2 at 90 days, no. (%)	50 (50.5)	54 (35.1)	22 (33.3)
Modified Rankin score of 6 at 90 days, no. (%)	24 (24.2)	59 (38.3)	22 (33.3)
Symptomatic ICH, no. (%)	4 (4.0)	7 (4.5)	5 (7.6)

## Discussion

This study primarily compared the safety and efficacy of three different thrombectomy devices (penumbra aspiration catheter, solitaire, and trevo retriever stent) in AIS patients with posterior circulation LVOs. Overall, the three mainstream thrombectomy devices were safe and effective in posterior circulation mechanical thrombectomy. The average TTR time was 47 min, 280 min for TOR, and 89.9% of the recanalization rate, with 50.5% of good prognosis rate and 24.2% of mortality rate. Penumbra aspiration catheter showed a significant advantage in recanalization time (32 min vs. 44 min and 41 min), though there was no statistically significant difference in 90-day functional outcomes (mRS 0–2) among device groups. This suggests a potential procedural advantage, but not clinical superiority. Functional recovery in posterior circulation stroke depends on various factors—including infarct location, collateral status, and patient baseline conditions—many of which may dilute the effect of faster reperfusion (16). Although our multivariate model adjusted for key variables (e.g., NIHSS, occlusion site, and TOR), residual confounding may still exist. Larger prospective studies are warranted to explore whether improved procedural efficiency translates into better long-term outcomes.

Previous studies have suggested aspiration or ADAPT/SNAKE techniques for anterior circulation AIS, which achieved a shorter procedure time for treatment of vessel recanalization (17–20). This study identified a significant advantage in the process time to recanalization for posterior circulation AIS, using the Penumbra aspiration catheter with comparable safety profiles. Similar to Gory et al. and Bernsen et al. (21, 22), our result indicates some level of superiority on the operational process related to the Penumbra aspiration catheter when it is used independently during mechanical thrombectomy. This may be related to the procedural simplicity of the aspiration catheter, since it does not cross the occlusion site, which may ultimately reduce device preparation time from first thrombus aspiration to re-aspiration.

Previous studies have demonstrated higher recanalization rates using stent retrievers compared to aspiration retriever devices, while surgical complication rates are higher after stent thrombectomy, especially related to embolic events (23). In this study, no significant difference related to successful recanalization rates or surgical complications was seen among devices. A similar prognosis was observed among the three thrombectomy device groups (22, 24–26). Lower baseline NIHSS, basilar artery occlusion, shorter TOR time, and absence of diabetes were significantly associated with improved functional outcomes at 90 days in this study, which was consistent with previous posterior circulation stroke studies^18-21^. As compared to anterior circulation stroke with LVOs, an overall lower rate of functional independence was found in posterior circulation stroke. Collateral circulation in anterior circulation strokes is well-developed, with the anterior cerebral artery (ACA) to middle cerebral artery (MCA) and posterior cerebral artery (PCA) to MCA collaterals. Posterior circulation collaterals are typically less mature, especially for “end vessels,” such as pontine perforators in the mid-basilar segment. These factors may explain in part the worse prognosis in patients with proximal basilar artery occlusion undergoing mechanical thrombectomy. Identifying an appropriate device choice and operational procedure could help reduce the operation and vessel recanalization time and improve the prognosis of AIS patients with posterior LVOs (27).

Although prior trials such as BASICS and BEST did not demonstrate a clear benefit of mechanical thrombectomy in posterior circulation stroke, these studies faced limitations, including delayed treatment, patient crossover, and under-enrollment, which may have influenced their conclusions (28, 29). In contrast, our study observed a higher rate of favorable outcomes (mRS 0–2 in 50.5%) and lower mortality (24.2%), which may reflect earlier reperfusion, more stringent patient selection, and higher recanalization success. These results are also in line with recent randomized trials such as ATTENTION and BAOCHE, which reported positive outcomes after mechanical thrombectomy in appropriately selected patients.

Several limitations should be acknowledged. First, the relatively small sample size from a single center limits statistical power and generalizability. This limitation also restricted our ability to perform formal subgroup analyses, interaction testing, or stratified comparisons based on device specifications (e.g., aspiration catheter diameter and stent retriever length) or occlusion site distribution. Second, as a retrospective, non-randomized study, selection bias and unmeasured confounding may have influenced treatment assignment and outcomes. Although multivariate logistic regression was used to adjust for known variables, including occlusion location and baseline characteristics, residual confounding cannot be excluded. Other device-related procedural complications—such as distal embolization, vessel perforation, or dissection—were not systematically recorded in the procedural dataset and therefore not analyzed. While no such events were explicitly documented in operative reports, their absence from formal records limits our ability to compare safety profiles across devices. Third, we excluded patients treated with multiple devices or rescue therapies to maintain methodological consistency. While these patients achieved successful reperfusion, their functional outcomes varied. Their exclusion may modestly bias overall outcomes toward less complex cases. Despite these limitations, our findings provide clinically relevant insights into procedural efficiency across commonly used thrombectomy devices in posterior circulation stroke. Future studies with larger, multicenter cohorts are needed to confirm and expand upon these observations.

Moreover, timely and effective reperfusion remains critical for patient outcomes, and challenges in post-procedural management and secondary prevention continue to warrant attention (30, 31).

## Conclusion

Penumbra aspiration catheter has a significant advantage over stent retriever-based therapies on the procedure time for posterior circulation LVOs. No significant improvement in the functional prognosis after aspiration mechanical thrombectomy for posterior circulation LVOs, as measured by mRS at 90 days, was noted compared to stent retrievers. The key factors for better functional outcome in posterior circulation LVOs include the absence of diabetes, lower baseline NIHSS, non-proximal occlusion, and shorter TOR time.

## Data Availability

The raw data supporting the conclusions of this article will be made available by the authors, without undue reservation.
